# Exploring early intervention in psychosis (EIP) perspectives towards pharmacogenomics (PGx) implementation to support antipsychotic prescribing: a multi-method qualitative exploration

**DOI:** 10.3389/fphar.2026.1837118

**Published:** 2026-06-10

**Authors:** A. Jameson, B. Fylan, G. S. Sagoo, C. F. Dalton, A. G. Cardno, J. Sohal, G. C. Bristow, S. L. McLean

**Affiliations:** 1 Department of Pharmacy, Bradford District Care NHS Foundation Trust, Saltaire, United Kingdom; 2 School of Pharmacy, Optometry and Medical Sciences, University of Bradford, Bradford, United Kingdom; 3 Wolfson Centre for Applied Health Research, Bradford, United Kingdom; 4 NIHR Yorkshire and Humber Patient Safety Research Collaboration, Bradford, United Kingdom; 5 Population Health Sciences Institute, Newcastle University, Newcastle, United Kingdom; 6 Biomolecular Sciences Research Centre, Sheffield Hallam University, Sheffield, United Kingdom; 7 Leeds Institute of Health Sciences, Faculty of Medicine and Health, University of Leeds, Leeds, United Kingdom

**Keywords:** antipsychotic prescribing, early intervention in psychosis, implementation, medicines optimization, pharmacogenetics, pharmacogenomics, qualitative research, reflexive thematic analysis

## Abstract

**Introduction:**

Pharmacogenomics (PGx) is an emerging medicines optimization tool that may support efficacy and safety relating to antipsychotic prescribing. Despite growing drug-gene evidence linked to antipsychotic response, how PGx could be implemented within Early Intervention in Psychosis (EIP) services, including the views of staff, service users, and carers remains underexplored.

**Methods:**

Semi-structured interviews with service users (n = 12), and online focus groups with EIP staff (n = 18), and carers (n = 3) across three National Health Service (NHS) EIP sites were analyzed using an integrative approach to reflexive thematic analysis.

**Results:**

Five themes and seven sub-themes were synthesized to describe stakeholder perspectives on implementing PGx to support antipsychotic prescribing in EIP services. Participants characterized EIP as a complex care ecosystem and described varying levels of understanding about PGx. Findings highlighted key implementation considerations, including when PGx should be offered, communication strategies, concerns about its integration into EIP pathways, and preferences for embedding PGx within routine care.

**Discussion:**

PGx was broadly perceived as an acceptable clinical intervention, analogous to established medicines-safety checks. However, implementation should prioritize shared decision-making, set realistic expectations about clinical utility, and be adequately resourced to avoid displacing other therapeutic approaches. This study complements existing drug-gene evidence by providing insights into clinical workflow integration, governance, and service design considerations specific to EIP contexts. As the evidence base for routine PGx use matures, its introduction in EIP services should be framed as a supportive, person-centered adjunct, and not a determinant of antipsychotic decision-making.

## 1 Introduction

Globally, psychotic disorders have a lifetime prevalence of around 1% (Moreno-Küstner et al., 2018). Although many psychotic episodes are brief and remitting, some persist and feature as part of severe mental health disorders, such as schizophrenia or bipolar disorder (Arciniegas, 2015). Around a half of patients who experience first episode psychosis (FEP) will achieve remission (Catalan et al., 2021; Lally et al., 2017). Early Intervention in Psychosis (EIP) services have emerged as a psychiatry sub-specialty (McGorry et al., 2008), aiming to support people experiencing FEP or early episode psychosis by offering interventions intended to help treat and manage psychosis and reduce future reliance on mental health (MH) services (Williams et al., 2025). EIP services are an established component of National Health Service (NHS) psychosis care in England and are nationally audited (Kendall, 2023). Pharmacological therapies are the most used intervention in the NHS (NICE, 2014) and in EIP services antipsychotic prescribing is common, being a key setting for antipsychotic initiation for many with FEP (Fusar-Poli et al., 2020; Jameson et al., 2024a). Antipsychotic prescribing is challenging, due to high rates of adverse drug reactions (ADRs), limited effectiveness, and low adherence rates (El Abdellati et al., 2020; Haddad et al., 2014).

Pharmacogenomics (PGx) studies how genetic variation can influence medication response (Qahwaji et al., 2024). Over 95% of the population possess at least one gene variant that impacts their response to a given medicine (David et al., 2021). PGx-informed prescribing is growing as an approach for medicines optimization, with the goal of improving outcomes by tailoring drug choice and dose to genetic data as part of a personalized approach to prescribing (Bousman et al., 2023; O’Shea et al., 2022). Several antipsychotics are linked with outcome variability as a result of PGx variants (Saadullah Khani et al., 2024). Such drug-gene interactions (DGIs) have implications for how antipsychotics exert their therapeutic action (pharmacodynamics) and how antipsychotics are handled by the body, from absorption, to distribution, metabolism, and elimination (pharmacokinetics) (Hernandez et al., 2024). Many antipsychotic DGIs relate to cytochrome P450 (CYP450) metabolism, particularly the CYP2D6 isoenzyme (Jameson et al., 2024a). Some CYP2D6 variants affect enzyme function resulting in abnormal metabolizer status, contributing to variable plasma levels of certain antipsychotics (Jameson et al., 2024a). Other DGIs can increase the risk of antipsychotic-induced weight gain (AIWG) and specific side effects such as extra-pyramidal side effects (EPSEs) or hyperprolactinemia (Hernandez et al., 2024).

Despite emerging evidence supporting the use of PGx in psychiatric clinical practice, its implementation globally has been limited, and only a few countries have implemented PGx in mental health settings (Bousman et al., 2023). Several factors hinder the clinical implementation of PGx in psychiatry, including a knowledge gap relating to PGx and uncertainty towards its use (Jameson et al., 2024b; Jameson et al., 2021). Despite these barriers, PGx use may improve the use of psychiatric medicines, including antipsychotics, by potentially enhancing safety, improving efficacy, and favoring cost-effectiveness associated with psychotropic prescribing (Sharew et al., 2024; Islam et al., 2021; Mason et al., 2026; Hernandez et al., 2024). Given the central role of antipsychotic prescribing in EIP care and psychosis management (NICE, 2014; Fusar-Poli et al., 2020; Jameson et al., 2024a), EIP services are a clinical setting of interest for PGx implementation to aid antipsychotic prescribing. Previous psychiatric PGx research has tended to focus on PGx use in the context of depression, and to date there has been limited exploration of implementation in the context of psychosis and EIP services (Jameson et al., 2024a; Bousman et al., 2023).

This study therefore aims to use qualitative methods to explore healthcare professional, service user, and carer perspectives towards the implementation of PGx in EIP settings, to support the prescribing of antipsychotics.

## 2 Methods

### 2.1 Study design

The study was designed as a multi-method qualitative exploration by incorporating multiple qualitative data generation approaches (both interviews and focus groups), conducted across three EIP sites, in three separate NHS MH trusts in the North of England. Sites were selected based on pragmatic constraints of the researcher. In the United Kingdom mental healthcare is delivered regionally as part of the NHS, but the vast majority of NHS MH trusts (regional organizations) commission an EIP service as part of their contract. As such, EIP services follow a standard approach to care, delivered locally but sharing key service offerings that are audited nationally (Kendall, 2023).

**FIGURE 1:**
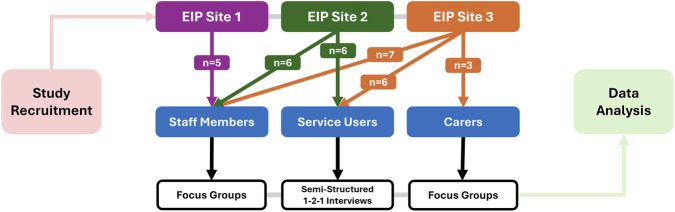
Study Activity Description: Overview of the study plan from recruitment to analysis, including sites and participant types from each site. EIP = Early Intervention in Psychosis.

Focus groups were conducted with professionals across all sites. Due to recruitment difficulties at one site, semi-structured interviews were conducted with service users from two of the three sites only. Carers in the carer focus group were recruited from one site only. Study activity across the participating sites can be seen in Figure 1. Focus groups were selected for staff and carer participants, to facilitate discussion of shared perspectives, generate interaction between participants, and capture collective views about PGx implementation. Interviews were selected for service user participants to provide a more private and supportive environment (see Section 2.5). No repeat interviews or focus groups were conducted.

Normalization Process Theory (NPT) (Mur et al., 2010) was used as a theoretical lens during different phases of the study. NPT was used to guide the development of the data generation tool. Separate topic guides for service user interviews, staff focus groups, and the carer focus group. Topics overlapped and included exploring perspectives towards the PGx implementation in EIP, understanding of PGx, and its potential for application during antipsychotic prescribing. Topic guides were developed by the researcher (AJ) and reviewed by the research supervisory team (SMc, BF, and GB) and the project’s patient and public involvement and engagement (PPIE) group (see Section 2.5).

AJ independently conducted the interviews and facilitated the focus groups, after attending several qualitative research training opportunities as part of their doctoral training. Service user and carer participants received a £20 voucher for participation, while EIP staff participated on a voluntary basis. Data generation occurred between September 2023 and February 2024, and was conducted remotely using Microsoft Teams (except one service user interview that was conducted using a telephone and a voice recorder). The mean length of service user interviews was 38 min (range 24–64 min). Transcription of interview and focus group transcripts was conducted by a university-approved external provider (see Section 2.4). This study was designed, conducted, and reported in accordance with the Consolidated Criteria for Reporting Qualitative Research (COREQ) checklist, and the completed checklist can be found in Supplementary Appendix 1 (Tong et al., 2007).

### 2.2 Participants, eligibility, and recruitment

Eligible service users were aged 18–65, under the care of an EIP service, with a past or present diagnosis of first episode psychosis (FEP), prescribed an antipsychotic, and assessed to have capacity to consent and participate in a safe manner. Antipsychotic-naïve and SUs aged under 18 years were excluded to minimize risk and ensure relevance. To be eligible, staff were required to be an EIP staff member, involved in either assessing, treating, or visiting EIP service users. Carers were eligible if they were a carer to a service user currently registered with an EIP service and involved in some aspect of the service user’s medicines pathway.

Eligibility criteria can be found in Table 1. Written consent was obtained from all participants. In total 33 participants were recruited for the study, including 12 service users, 18 professional participants involved across four separate focus groups, and 3 carers in a single focus group. Staff focus groups were organized per study site, with an additional fourth, mixed site focus group being conducted for staff who could not attend the focus group for their site.

**TABLE 1:** Eligibility criteria.

​	Inclusion	Exclusion
Service user	• Aged 18–65 • Under the care of an EIP service • Current or previous diagnosis or working diagnosis of first or early psychosis • Currently or previously prescribed an antipsychotic • Assessed by a member of their clinical team to have capacity to consent and sufficiently stable current mental state	• No current or previous diagnosis of psychosis • Service users in child and adolescent mental health (CAMHs) EIP services • Lacking capacity to consent to participate or take part in the study • Newly referred service users to the EIP service who are yet to receive an initial consultation
Carer	• Carer to a service user currently under the care of an EIP service • Involved in the medicines pathway for the individual they care for (e.g., administering, managing, monitoring, or discussing antipsychotic treatment)	• Carer of service user not registered with or cared for by an EIP service • Not involved in any aspect of the medicines pathway for antipsychotics for the individual they care for
Staff	• Registered member of staff with an EIP service involved in either assessing, treating, delivering interventions, or visiting service users under the care of the EIP team	• Not working in a patient facing role (e.g., administrative roles) • Not working for an EIP service • Not involved in patient care within an EIP service

Description: Inclusion and exclusion for each participant group. EIP, early intervention in psychosis.

A stratified purposive sampling approach (Palinkas et al., 2015) was used to include participants who could provide relevant perspectives. Recruitment was purposive in seeking groups (service users, staff, and carers) most involved or affected if PGx becomes available in EIP settings. Service users were approached by a key member of their clinical team, while staff and carers were approached through existing professional networks either face-to-face or via email. The sampling approach was also stratified to, where feasible, ensure the sample was representative of the diversity in age and ethnicity among service users and job roles among EIP staff. Sample size was guided by principles of information power (Malterud et al., 2016), that proposes the volume of data necessary for a study is dependent on the study aims, sample specificity, use of established theory, data richness, and analytic approach.

### 2.3 Data analysis

In order to assess the breadth, depth, and diversity of the accounts emerging from different participant groups and study sites, the researcher regularly reviewed the data during the data generation phase by using reflective field notes and ongoing familiarization with transcripts. This helped to determine when to stop recruitment, when the dataset was judged to provide sufficiently rich coverage of the research questions across the participant groups and data generation methods. A limitation to this approach was the low recruitment of carers, which reduced the relative information power of the carer dataset, placing constraints on the extent to which carer perspectives could shape theme development during the analysis. Limitations of the study in relation to information power are noted in the discussion.

**FIGURE 2:**
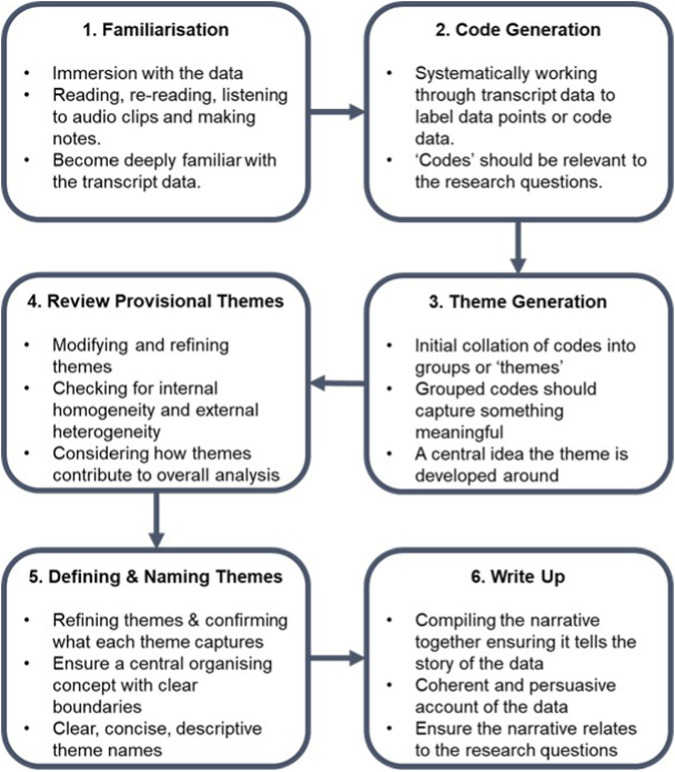
Stages of reflexive thematic analysis (RTA) Description: Stages of RTA as adapted from (30).

Reflexive thematic analysis (RTA) (Braun and Clarke, 2006; Braun and Clarke, 2019) was used to analyze all interview and focus group transcripts in a single, integrative analysis across service user, carer, and EIP staff data. Following the six phases of RTA (see Figure 2), the researcher iteratively moved between familiarization, open coding, theme construction, review, theme definition, and write-up (Byrne, 2021), using an analytic integration approach to ensure multivocality and coherence across insights from the participant groups and across the different data types (Tracy, 2010; Cronin et al., 2008). NVivo by Lumivero (2026) was used to support data management following initial by-hand coding. To enhance rigor, BF independently coded early transcripts and held debriefs with AJ to interrogate coding decisions, define theme boundaries, and to ensure internal homogeneity and external heterogeneity of themes (Patton, 1990).

Although Normalization Process Theory (NPT) (Mur et al., 2010) informed topic guide development, it was not imposed on the data analysis to avoid constraining inductive theme generation. NPT was instead subsequently used to interpret and situate findings following data analysis. The researcher maintained a reflexive stance throughout (Braun and Clarke, 2019), being aware of how their background as a male clinical pharmacist and PhD researcher, and how this may shape interpretations during analysis. The reporting of themes followed recently published guidance for conducting RTA (Braun and Clarke, 2024).

### 2.4 Ethics

Ethical approval was granted by the NHS Health Research Authority and Health and Care Research Wales on 27/04/2023 (REC 23/PR/0,260) following internal university review and attendance at the London-City and East Research Ethics Committee (REC). Capacity and capability were confirmed by local research departments for each participating site. Inclusion procedures required staff at participating sites to approach service users (SUs) only if they were judged to have capacity and sufficiently stable mental health to provide informed consent. All participants were provided with approved participant information sheets to enable prospective participants to make informed decisions. Following written consent, service user interviews and staff and carer focus groups were conducted using Microsoft Teams. Recording of data generation took place in line with procedures outlined in the consent form. Transcription of audio recordings was conducted by an external university-approved provider adhering to a pre-defined data sharing plan that ensured privacy and data protection provisions.

### 2.5 Patient and public involvement and engagement (PPIE)

A PPIE group contributed to the study across design, conduction, and interpretation phases. They advised on using one-to-one interviews as a data generation methods for service users and provided input co-designing study materials and a PGx FAQs sheet. These activities improved accessibility, clarified what PGx is, and emphasized PGx is not a diagnostic or prognostic intervention. They also suggested a carer focused recruitment strategy and advised on reimbursement levels for participants. Although participants themselves did not, the PPIE group reviewed early coding and theme generation and offered interpretative feedback.

## 3 Results

### 3.1 Participants

Overall 33 participants (10M, 23 F) contributed to this study and an overview of participants can be found in Table 2. A total of 12 service users participated via one-to-one semi-structured interviews, 18 EIP staff members across four focus groups, and 3 carer participants in one carer focus group. Participants were from a range of ethnic backgrounds and were aged from 21 to 56 years old. There was a relatively equal mix of service user participants by sex. Staff members varied by professional background, covering disciplines such as medical psychiatry, nursing, psychology, and EIP support roles.

**TABLE 2:** Participant characteristics.

Participant Type	Study IDs	Age	Sex	Ethnicity	No. of trialled antipsychotics/job roles
Service users	ID1 – ID12	• < 21 (n = 1) • 21–30 (n = 5) • 31–40 (n = 5) • > 40 (n = 1)	• Male (n = 5) • Female (n = 7)	•White british (n = 7) • British asian (n = 3) • Mixed black and white british (n = 2)	•1 (n = 3) • 2 (n = 4) • 3 (n = 3) • 4 (n = 2)
Staff	FG1 – FG4	• 25–30 (n = 3) • 31–40 (n = 6) • 41–50 (n = 6) • 51–60 (n = 3)	• Male (n = 5) • Female (n = 13)	• White british (n = 16) • British asian (n = 1) • White european (n = 1)	• Mental health nurse (n = 5) • Psychiatrist (n = 4) • Psychologist (n = 3) • Cognitive behavioural therapist/Psychotherapist (n = 2) • Care coordinator (n = 1) • Occupational therapist (n = 1) • Nursing associate (n = 1) • Support worker (n = 1)
Carer	FG5	• 41–50 (n = 1) • 51–60 (n = 2)	• Female (n = 3)	• White british (n = 2) • British asian (n = 1)	• N/A

Description: Overview of study participants, their sex and ethnicity, and job role (staff) or number of trialled antipsychotics (service users).

### 3.2 Overview of themes

**FIGURE 3:**
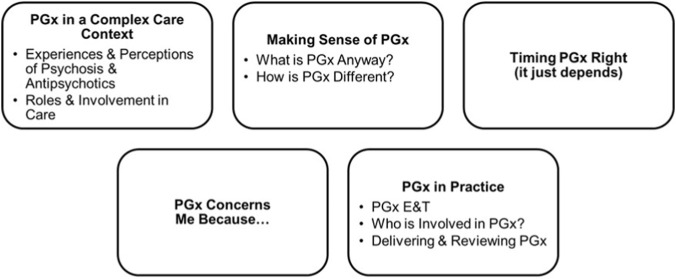
Reflexive Thematic Analysis Themes and Sub-Themes Description: Themes and sub-themes generated during the reflexive thematic analysis. PGx = pharmacogenomics; E&T = education and training.

The Reflexive Thematic Analysis (RTA) generated five main themes and seven sub-themes that capture participant experiences of EIP care, their perspectives towards PGx implementation in EIP settings and their conceptualizations of PGx implementation. Figure 3 shows the main themes and sub-themes. Supplementary Appendix 2 contains a comprehensive list of data extracts from the RTA.

#### 3.2.1 Theme 1 “PGx in a complex care context”

Staff participants described how EIP services deliver care within a highly complex system, marked by variability in clinical standards, service delivery pressures, and challenges. Service users and carers had mixed experiences of psychosis and differing views towards antipsychotics were articulated. They thought that PGx implementation would inevitably be shaped by these pre-existing complexities and views.

**Sub-theme:** Experiences and Perceptions of Psychosis and Antipsychotics.

Service users reflected upon their psychosis journeys, describing multifaceted experiences as if ‘somebody else had taken over’. Their experiences and beliefs towards use of antipsychotics were mixed, with some viewing them as useful in psychosis recovery, while others questioned long-term benefits. Issues relating to antipsychotics were highlighted, including side effects, the trial-and-error approach to prescribing, and issues of non-adherence. Staff recognized the problems associated with trial-and-error prescribing, citing ‘there’s no strong evidence that one’s any better than any other’ and emphasized the difficulties of prescribing, including lack of prescribing guidance.

“They put me on 600 mg […], and […] it did help pretty quickly, but I could not cope with the side effects. And then I tried to lower myself down to 200, but I could feel everything starting again. So, the 400 seems to be the right place for me at the moment” [ID9, White British Male]

**Sub theme:** Roles and Involvement in Care.

Staff described the multidisciplinary nature of EIP care and discussed their role in service delivery, highlighting work pressures such as understaffing and time constraints. These pressures were perceived to impact future PGx implementation and delivery. Trust and communicating information were central to EIP teams, as service users described that ‘when you’re talking to somebody familiar, that tends to be more helpful’ *[ID2, White British Male]*. Service users reflected on empowerment through shared decision-making, and underscored how PGx could support this if they trusted information about PGx. Carers facilitated medication administration and monitoring, and in some cases were ‘in charge of giving medication’ *[FG5, Carer, White British Female]* at home, highlighting the key role of carers in medicines and liaison with EIP teams.

“Once I've got more information about this [PGx], then maybe I can go ahead with it. Once I believe that I can actually trust this program, then yeah” [ID10, British Asian Male]

#### 3.2.2 Theme 2 ‘making sense of PGx’

Understanding of PGx varied, generally being perceived as new but with similarities and differences to current practice. Perceptions, acceptability towards, and perceived potential benefits of PGx were articulated, in addition to uncertainties held about PGx. Participants viewed PGx as distinct from other genetic tests and that overall PGx aligns with the values of EIP services.

**Sub-theme:** “What is PGx Anyway?”

Although some staff, particularly psychiatrists, had heard of PGx or at least knew of the concept, generally participants described PGx as ‘new’ and the study as the ‘first time’ they had heard of it. As understanding developed, PGx was seen by most as potentially valuable to personalize prescribing, as one service user explained ‘I’d be intrigued to find out what medication I would be prescribed if I had a PGx test’. Staff felt PGx can support more informed antipsychotic switching and dosing, and be useful for minimizing ADRs. Some expressed it may improve adherence, reduce recovery time, and shorten hospital stays. Others felt it could shift perceptions about medicines and promote a more collaboration during recovery.

“We go through so much trial-and-error […] because it's a guessing game until we’re hitting it on the head. If we could get there quicker, minimize side effects, I am sure we'll have much better outcomes, better take up and consistency with the medication. I think [PGx] is a really good idea.” [FG1, Nurse Associate]

**Sub-theme:** ‘How is PGx Different?’

Across participant groups, PGx was not perceived as radically different from existing EIP practice, with staff questioning ‘how big a thing does [PGx] need to be?’ and ‘what is the difference between [PGx] and information from blood tests?‘. Participants believed PGx can be positioned in line with routine blood tests and physical health monitoring, as an additional safety measure. However, for service users, distinguishing PGx from other types of genetic testing was emphasized, to explain that PGx examines drug-gene interactions only, without diagnostic or prognostic implications. With PGx consent, staff drew parallels to how consent is needed for routine clinical tests. Although PGx was viewed as conceptually aligned with EIP values, it was still seen as a new approach to delivering care in the context of antipsychotic prescribing.

“I think it’ll be worthwhile to let the patient know that it does not test their whole genetic profile, […] that does not feel quite as potentially invasive.” [ID2, White British Male]

#### 3.2.3 Theme 3 “access to and timing PGx right (it just depends)”

Participants emphasized that timing of PGx offering should be tailored to an individual’s mental state, as learning that genetics is being used to tailor care has the potential to ‘set people off worse or make someone’s psychosis worse’ *[ID12, White British Female]*, if offered during acute psychosis. Therefore, offering PGx during acute psychosis was deemed potentially inappropriate due to impaired decision-making capacity and risk it could worsen psychosis symptoms. PGx was therefore preferred following some initial recovery from acute psychotic symptoms, when information can be delivered and understood accordingly, and when consent for PGx can be appropriately obtained.

“I probably feel like [PGx] would not be suitable for someone that's in the middle of a psychotic episode because they would not be able to process what's happening or what they're dealing with.” [ID6, British Asian Female]

Staff favored PGx later in the EIP pathway, once a therapeutic relationship is established with service users. Staff were also in support of preemptive PGx (before antipsychotic initiation) to tailor prescribing. Integrating PGx within existing physical health clinics was proposed, as ‘that’s people’s routine physical checks’, which could offer a flexibility for PGx implementation, both during baseline antipsychotic monitoring or later during antipsychotic reviews. Staff views about access to PGx were varied, some wanted universal availability to avoid inequity and ensure quieter service users are not overlooked. While others were cautious about routine testing, noting that not all service users would want to have PGx, nor clinically require it.

“I would not want it to be implemented as a standard […] for everyone in EIP […]. There are those people who do not want to know certain things or, you know, do not ever want to try medication” [FG4, Mental Health Nurse]

#### 3.2.4 Theme 4 “PGx concerns Me because … ”

All participant groups identified ethical issues including PGx data storage and third-party access, with the potential for misuse or discrimination being noted by global majority ancestry groups in particular. Staff anticipated challenges obtaining consent for PGx and questioned how this would work for detained services users or those ‘lacking capacity’. Staff also cautioned against an overreliance on PGx, citing it could replace clinical judgement if prescribers become ‘bypassed’. Other risks were noted including PGx reports deskilling prescribers, delaying treatment while awaiting results, or being misinterpreted. Some doubted whether PGx would narrow treatment options and that it may add to the trial-and-error prescribing approach.

Some staff feared that PGx could further medicalize psychosis and contradict EIP’s biopsychosocial approach. Staff also highlighted practical concerns including added workload, cost, and risk of confusing staff and service users if PGx is poorly communicated. Existing constraints within EIP services were believed to make PGx implementation more challenging. A minority of service users though did report no concerns, perceiving PGx as a ‘safer’ route to more tailored prescribing.

“We just need to make sure that we’ve still got that broad view in early intervention, holistic care, of different things, so that it [PGx] does not over-emphasize biology” [FG2, Consultant Psychologist]

#### 3.2.5 Theme 5 ‘PGx in practice’

Participants described how PGx could be prepared for, delivered, and evaluated within EIP. Views centered on building staff capability to deliver PGx (PGx Education and Training), clarifying who should deliver PGx services (Who is Involved in PGx?), and finally the practicalities providing PGx services including counseling, consenting, sampling, reporting, and utilization (Delivering and Reviewing PGx).

**Sub-theme:** PGx Education and Training.

Staff wanted role-specific, face-to-face training to build confidence with PGx, its scope and limitations, and the practical steps involved in PGx like consenting, counseling, interpretation (for prescribers), and IT integration. Engaging formats, like role play, were preferred compared to passive emails or online materials. SUs emphasized that PGx must be explained by knowledgeable and appropriately qualified professionals.

“We’d need […] training on the interpretation of the results, depending on what format they are given to us. That’ll be useful […] to have some training on what it means and how accurate things are, so we can counsel patients correctly.” [FG3, Consultant Psychiatrist]

**Sub-theme:** Who is Involved in PGx?

Psychiatrists were seen as pivotal for explaining PGx in the context of antipsychotic selection, dose, and ADRs, and were perceived to have overall ‘professional responsibility’ for PGx. Care coordinators and support workers roles were viewed as important for introducing PGx and supporting decision-making, due to established relationships and perceived trust of service users. Pharmacists were also identified as important for ensuring PGx safety, counseling, candidate identification and clinical decision support, as PGx was perceived to ‘fit within the realm of pharmacy’. Carers were considered well-placed to contribute and support discussions and decisions around PGx.

“Because [carers] they’re the people that are best placed […] to also be involved in decision making. Even though our young people are adults, they’re not really in the best place at that time.” [FG5, Carer, British Asian Female]

**Sub-theme:** Delivering and Reviewing PGx.

Participants wanted clear, and concise information and counseling about PGx, using leaflets, flowcharts, videos, and evidence summaries, using accessible language to avoid ‘rendering people numb’ *[FG2, Consultant Psychiatrist]*. Choice and consent for PGx was an expectation, with the potential for ‘best interests decisions’ *[FG4, Clinical Psychologist]* to be made by staff. Saliva sampling was preferred over blood samples as its ‘less awkward and *painful’ [ID2, White British Male]*, and staff wanted turnaround time in days to weeks. They also believed that results, and any associated prescribing changes, should be communicated in person with simple visual aids or written reports. Interoperability of PGx results across providers (e.g., GPs and pharmacies) was desired, with participants wanting results storage within NHS systems and no third-party sharing. Staff requested PGx outcome data collection and review of PGx implementation to use quantitative and qualitative data. Reflective practice to improve implementation was viewed as important to learn and enhance PGx services.

“Knowing the basics about what PGx is and how it might be able to help improve the quality of care when you're on medication […].” [ID4, British Asian Female]

“Stored with the GP and […] EIP too. Because the whole point of the testing is to get more accurate … a more effective treatment.” [ID2, White British Male]

## 4 Discussion

The study offers an integrated account of how EIP stakeholders make sense of PGx and what is required for successful implementation. Developed through a reflexive lens, participants positioned PGx as a potential medicines safety tool, and highlighted the potential of PGx to support person-centered care, shared decision-making (SDM), and reduce avoidable harms (e.g., ADRs). Participants recognized PGx is not a magic bullet for uncertainties associated with antipsychotic prescribing and specified risks including reinforcing the medical model of psychosis, widening existing inequalities, and detracting from psychosocial care approaches. These insights are consistent with the PGx implementation literature (Chan et al., 2017; Jameson et al., 2024b) and situate PGx as an acceptable and potentially helpful intervention. Our findings add to the literature by suggesting implementation factors specific for EIP settings, including sensitive timing of PGx in the context of psychosis, embedding PGx into existing clinical workflows, clear communication around PGx, role-specific PGx training and data safeguards.

Experiences of psychosis and EIP services were characterized by complexity and perceived usefulness of antipsychotics was variable, reflecting the trial-and-error reality of prescribing. PGx perceived benefits included improved confidence, trust, and therapeutic alliance between service users and prescribers. In this context, PGx was viewed as an additional datapoint, like other safety precautions, rather than a single determinant of prescribing decisions. Critically, participants anticipated PGx as an information resource that could deepen antipsychotic prescribing discussions. This suggests PGx may help remove uncertainty by addressing known key factors, including adverse effects, autonomy, and knowledge, during antipsychotic prescribing (Kaar et al., 2019). SDM has documented challenges (Haugom et al., 2020; Shepherd et al., 2014). Yet our findings suggest PGx could act as a bridge between service users and practitioners, highlighting PGx has potential difficult to measure benefits beyond clinical outcomes.

Participants urged caution about expectation setting for PGx. While some perceived PGx as promising innovation that may reduce trial-and-error, there were also identified risks associated with overselling PGx, disappointment from PGx results, and medicalization of care. Concern about overreliance on genetic factors of antipsychotic response (Warner-Levy et al., 2026; Soria-Chacartegui et al., 2021; Saadullah Khani et al., 2024) and overlooking non-genetic factors, such as social determinants and trauma (Stilo and Murray, 2019), was raised. This reflects wider complexity of psychotropic prescribing and how it is highly preference sensitive and context dependent (Yager et al., 2022; Roberts et al., 2018). Participants therefore valued plain language explanations, distinguishing PGx from diagnostic or prognostic genetic testing and emphasizing PGx as a support tool. This resistance to genetic determinism (Harden, 2023) mirrors current guidance for using PGx during antipsychotic prescribing (Baldacci et al., 2023; Beunk et al., 2024), where recommendations are selective and drug-specific rather than universally suggested. PGx should not replace the need for ongoing review and assessment as part of a holistic approach to psychosis care. With this view, PGx aligns with normalization process theory, and stressing coherence work to understand what PGx is and what it is for to ensure successful implementation.

Timing of PGx was ethically and practically important for participants. A preemptive, interoperable PGx testing model was attractive for minimizing prescribing delays and optimizing usefulness. However, participants questioned introduction of PGx during acute psychosis, due to potential limited capacity of service users and paranoia that language about genetics could contribute to. A flexible approach was preferred, offering PGx once rapport had been established and revisiting PGx after key clinical events (e.g., following ADRs or during treatment changes). This idea of timing PGx complements the wider psychosis literature exploring the importance of therapeutic alliance, trust, and relational continuity when making treatment decisions. Previous studies have shown that SDM in psychosis is often reliant on developing trust and engagement between practitioners and service users (Haugom et al., 2020; Brown and Parry, 2024; Browne et al., 2019). In this sense, PGx may be more appropriate when service users can meaningfully engage with it, rather than at the earliest stage a PGx test can technically be offered.

Concerns about PGx extended to issues of consent and capacity, how these would be navigated, and when ‘best interests’ decisions are appropriate. Concerns also covered data governance and where PGx data is stored or shared. These concerns reflect the wider debate in the psychiatric genetics literature, where privacy, potential discrimination, and psychological impacts of genetic information have been identified as barriers to implementation (Pinzón-Espinosa et al., 2022; Ioannou et al., 2024). In EIP, these issues may be amplified due to the stigma of psychosis and the sensitivities of mental health genetics. Participants also highlighted concerns over ancestry bias in PGx evidence and potential impacts on practice such as deskilling prescribers, treatment delay, and overshadowing psychosocial interventions. Addressing these concerns through co-designed safeguards may support implementation and strengthen confidence in rollout by facilitating the relational and organizational work required to routinely embed PGx.

Staff desired role-specific training, with prescribers needing more in-depth, hands-on training compared to non-prescribing staff that prefer training to gain confidence in discussing PGx. Similar needs and skills gaps have been widely reported in the PGx literature where clinicians report enthusiasm towards PGx but cite uncertainty about clinical utility, results interpretation, and workflow integration (Keeling et al., 2021; Alghamdi et al., 2025). Pharmacy was seen to have a potential role in PGx counseling, results interpretation, and PGx-informed medicines optimization, reflecting wider advocating for multidisciplinary PGx delivery (Maruf and Aziz, 2023). Simple PGx reports were preferred to anchor results in SDM by framing PGx as informative rather than directive. These findings emphasize the need to clarify roles, build capability, and embed accessible decision support to enable the collective action work required to operationalize PGx and help teams undertake the practical work needed to integrate PGx.

Interoperability between care providers was seen as essential for implementation, highlighting a need for top down governance to oversee wider implementation efforts. The role of the NHS Genomic Medicine Service (GMS) in PGx rollout therefore remains important (Rafi et al., 2020). The results presented broadly align with wider PGx implementation literature that suggest common barriers like mixed clinical utility, lack of infrastructure, and limited workforce readiness exist (McDermott et al., 2025; Alghamdi et al., 2025). Our findings offer further insights, that are specific for psychosis pathways and complement findings from research conducted exploring PGx integration in behavioural health clinics (Sharma et al., 2024).

### 4.1 Limitations

The study had several limitations, such as the poor exposure participants had to PGx prior to the study. This meant that in order to meaningfully participate in the study, participants were required to receive introductory information about PGx which may have shaped their early judgements, despite an effort to keep resources neutral. Opinions expressed were therefore purely hypothetical, in that none of the participants had undergone PGx testing and had no personal experience to draw on. Several factors reduced the information power of the study sample, including limited carer recruitment, one study site being unable to recruit service users, and urban geographic location of the three sites within a 40-mile radius. These factors reflected recruitment difficulties and limited transferability of the findings across all EIP sites. Although purposive sampling aimed for sample diversity, staff participants were predominantly White British and the sample size was modest. Nonetheless, the use of an established theoretical framework, integrative analytic approach, and a diverse multi-stakeholder sample provides a basis for transferable, theoretically informed insights that are likely to resonate across comparable mental health and psychosis care contexts.

### 4.2 Future directions

Future research should aim to co-design a minimally viable PGx pathway in EIP that integrates the latest international guidance for PGx-informed antipsychotic prescribing. Given the emerging clinical opportunities for pharmacists in the NHS, and increasing adoption of PGx by pharmacy professionals globally, the role of pharmacists in EIP and psychiatric PGx implementation should be explored further. This integrative analysis offers insightful suggested starting points for PGx implementation in EIP services, including role clarification, simple reporting of PGx results, integration within electronic health records, and consideration for adapting the timing of PGx introduction to an individual’s psychosis journey. More research is needed to adapt PGx implementation from specific specialities where uptake has progressed faster (e.g., oncology and cardiology). Outcomes from the PROGRESS study will hold value for wider implementation in the United Kingdom (Catalan et al., 2021). Further research about the cost-effectiveness of when to offer PGx is also needed.

## 5 Conclusion

This study found that EIP stakeholders are open to PGx implementation when it is positioned as a supportive aid that is ethically delivered and is relational to standard practice and approaches. Implementation should be mindful in what PGx promises and accurately communicate the potential benefits and limitations of PGx. Implementation efforts should aim to time the use of PGx carefully and embed PGx within the existing EIP care infrastructure. Although PGx has the potential to reduce avoidable harm and support more appropriate antipsychotic prescribing, it should not replace clinical judgement, nor the psychosocial efforts underpinning modern approaches to psychosis recovery. As evidence about PGx-informed antipsychotic prescribing matures, implementation should be pragmatic and adaptable based on new emerging evidence. Any PGx implementation in EIP settings should be evaluated to offer a more credible and real-world insight into the potential outcomes, building on the findings generated by this study.

## Data Availability

The original contributions presented in the study are included in the article/Supplementary Material, further inquiries can be directed to the corresponding author.
