# Supplementary material for: Exploring early intervention in psychosis (EIP) perspectives towards pharmacogenomics (PGx) implementation to support antipsychotic prescribing: a multi-method qualitative exploration
Source: Front Pharmacol. 2026 Jun 10;17:1837118. doi: 10.3389/fphar.2026.1837118 (PMC13290928; doi:10.3389/fphar.2026.1837118)
Supplement: Supplementary file 1 [file DataSheet2.pdf]

Supplementary Material A:
Reflexive Thematic Analysis Data Extracts by Theme and Sub-Theme

**Theme 1: PGx in a Complex Care Context**	
Sub-theme: Experiences and Perceptions of Psychosis and Antipsychotics	
"I was unwell and full of stress. I think the stress led to me getting really worked up, and I felt like I wasn't myself, I felt like somebody else had taken over. I was no longer [name]. I was a completely different person"	ID10, British Asian Male
"When a person is psychotic, there can be all wild ideas that are going through their minds […] It feels like […] you're just vomiting up all your memories, thoughts, and experiences that you've accumulated over the course of your life. And then it's all being bashed up and remixed and psychosis"	ID2, White British Male
"There are loads of, I guess, confounding factors, aren't there? There's a lot of things at play and I kind of always describe it to patients as like a puzzle and medication is only really one piece and everything else needs to come together to make it work"	FG4, Care Coordinator
"You could end up thinking there's the magic drug out there, 'oh, wow, well, can I have some of those [PGx] tests, please?' And chasing the magic drug at the expense of, what else is going on in your life? […] 'there's the magic pill at the end of this long tunnel, and now we're going to really get you there through these genetics'. I wonder if that, is in some people's minds"	FG2, Consultant Psychiatrist
"What I've tended to find myself doing is a lot of dose adjustments or tapering or cross-tapering or switching medicines to try and find something a bit more optimal or acceptable for a client or for a person"	FG2, Mental Health Nurse
"I've had quite a few conversations with people around sort of the impact of medication and them being able to engage in therapy how they want to. They start struggling with a numbing of emotions […] not really being able to engage with conversation or the emotions that are associated with what they're talking about"	FG4, Clinical Psychologist
"They put me on 600 milligrams straight away, and I guess it did help pretty quickly, but I couldn't cope with the side effects it was causing. And then I tried to lower myself down to 200, but I could feel everything starting again. So, the 400 seems to be the right place for me at the moment"	ID9, White British Male
"He realised that it was actually doing him some good, but then he hated the fact of what it was doing to his body, the fact he was so hungry all the time, he couldn't stop eating and he just couldn't wait to be off them really"	FG5, Carer, White British Female
"With antipsychotics we have no guidance really into what is first line, second line, third line, […] it's been mentioned that there's no strong evidence that one's any better than any other"	FG1, Advanced Nurse Practitioner
"Getting blood out of service users and encouraging them to attend their physical health appointments is quite challenging sometimes"	FG1, Support Worker
"It was a real struggle initially. It was really hard to get him to take any medication at all so, […], you're the carer so you're in charge of all that and obviously when they're in an episode, they think the medication is harmful towards them so, it's that thing of them not taking it so, he did that a lot so, it would be I was in charge	FG5, Carer, White British Female

of giving him the medication and he would just stick it in his cheek and then spit it out and it would take a little while because it's that thing of, oh, it's not working"	
Sub-theme: Roles and Involvement in Care	
"I show them a table where they can see their side effect profile of the most commonly prescribed second generation antipsychotics, so they have that understanding that it is likely that there would be side effects and it's more about the choice, so the effectiveness is quite similar in all antipsychotics but the choice sometimes comes down to what could be the more tolerable side effects should you have side effects so that conversation sometimes takes quite a bit of time"	FG1, Consultant Psychiatrist
"I would be involved in medical reviews with the doctor and then I suppose I would monitor effects, side effects, responses to medications. I'd involve a doctor if I had concerns or if I felt that things needed tweaking. Yeah, I'd just kind of oversee it, I suppose."	FG4, Care Coordinator
"I suppose it's just being careful when you're giving information to someone. So, when you are getting them [PGx] results back it's like, 'Do they really need it? Can't you get a husband or someone if they've got, next of kin.' Because it's not going to make much difference when you're in that acute stage."	ID12, White British Female
"My involvement then is maybe liaising with the care coordinator and medics as well as with the service user, but working more on, I guess, the... I would say that the psychological side of taking the medication, as well as what it means to that person and their feelings around it and how they're managing that"	FG1, CBT Therapist
"When I first went to hospital and the psychiatrist said, well, we'll just consult the pharmacist. I couldn't believe that the psychiatrist didn't know that, but then thinking about it now, I realise that medicines change, and the pharmacist has a very, very important role to play in it"	FG5, Carer, White British Female
"I don't offer my patients all the antipsychotics when I'm starting because they just get bamboozled when there's loads, right? I might give them a choice of just two"	FG3, Consultant Psychiatrist
"I don't really feel involved at all to be honest with that, because it feels like I'm talking to a brick wall sometimes."	ID5, Mixed Black and White British Female
"I was given all the information from day one, and they were helping and explaining stuff rather than just saying, 'You need this medication. This will help you.' they explained everything. It took that fear away"	ID9, White British Male
"I: And who would you want to communicate these [PGx] results? P: Probably somebody that I've built a rapport with, like as the care coordinator. Because that's somebody who I see a lot of. And I think when you're talking to somebody who's familiar to you, that tends to be more helpful. […] Probably someone, yeah, somebody familiar"	ID2, White British Male
"Once I've got more information about this [PGx], then maybe I can go ahead with it. Once I believe that I can actually trust this program, then yeah"	ID10, British Asian Male
"There's obviously a lot of really complex concepts and science behind this [PGx]. I suppose, yes, it's about how to distil it in a way that can be easily communicated, and also, that… Not just portraying the benefits of this, that it is a complex situation about… There's pros and cons to this, and how to then have those conversations with the, yes, the people in front of you."	FG2, Consultant Psychologist

Theme 2: Making Sense of PGx	
Sub-theme: What is PGx Anyway?	
"It [PGx] is new to me. I don't know what that word was before. I never come across it before. Today is the first time it's been mentioned to me."	ID10, British Asian Male
"I'm not against sort of it [PGx] being implemented, but I just wonder, in an already fairly medical system, not necessarily EI, but particularly the places where people come to us from i.e., hospital. To then sort of start with us and have quite a medical procedure. It might feel yeah, like a continuation really of medical models, which I think sometimes we work in EI to challenge with people and to think about how their life can be that can be more than 'illness'."	FG4, Mental Health Nurse
"I think I'd be intrigued to find out what sort of medication would be prescribed if I had a pharmacogenetic test."	ID6, British Asian Female
"I was just going to say that these things in 10 years' time, this might be the most normal thing everybody's doing so it'll be the same as a blood test, the same as taking a full blood count."	FG1, Consultant Psychiatrist
"The only thing I would say is I think the terminology, which I think is very important, that the name, it sounds a bit to somebody who's in psychosis, you could think that they might think well, this is another thing, I don't know. It's just the name of it. It's not scary. It's just that it's…It could be misconstrued by somebody that was misconstruing a lot of stuff that they're seeing and thinking."	FG5, Carer, White British Female
"We go through so much trial and error sometimes with antipsychotic medication which can't be avoided because it's a guessing game, isn't it, until we're hitting it on the head. If we could get there quicker, minimise people's side effects, I am sure we'll have much better outcomes, much better take up and consistency with the medication. I think [PGx] it's a really good idea."	FG1, Nurse Associate
"They should introduce pharmacogenomics. It is going to make people with my sort of condition, my sort of history, it will help enable them to get treatment which will suit them better rather than just like what they did with me before was like trial and error."	ID10, British Asian Male
"I think side effects would be more useful. I mean the metabolising, yes, would be useful but I think the most useful would be something that tell us if somebody is going to have propensity to weight gain because that's the biggest, or high, of sedation perhaps, if they're going to be overly sedated with smaller amounts of antipsychotic."	FG1, Consultant Psychiatrist
"I'd probably say before because it would reduce the possibility of experiencing side effects, if it's more likely to match your DNA, so your body is more likely to not react negatively to taking medication."	ID6, British Asian Female
"I think [PGx] it could be really useful. And it saves a lot of that trial and error, and if it can make people better quicker and things like that."	ID11, White British Female
"I: What impact, if any, do you feel that pharmacogenetic testing could have had on how involved you felt in the decision making that went into you being prescribed an antipsychotic? P: I guess I would've felt more involved because we're talking about an aspect of my own genetic profile. I would feel like I was more involved with it."	ID2, White British Male

"It makes you think, what do we really mean, and what is meaningful shared decision-making? I think with something like this [PGx], it could open the door to some better conversations about people's relationship to these treatments, and their ideas about it or their beliefs about it, what they want, what they hope for, what they're fearful of. That would be my hope"	FG2, Psychotherapist / Family Therapist
Sub-theme: How is PGx Different?	
"I had it when I was under neurology, they did genetic tests. She just said, 'Oh we can do genetic testing, this, that, the other'. It was just a normal conversation. It wasn't overthought and she said, 'I'll let you know if there's anything with results.' It was quite normalised. The thing, whether we do it that way, just like 'this is what we usually do', type thing, it's how we sell it."	FG3, Occupational Therapist
"How big a thing does it [PGx] need to become? Does it need to be, if we do it, does everybody need to be emailed and know about this, or actually, can it be quite a streamlined thing, that this is just an adjunct to a decision, a pathway, a medication pathway, that if we're going for medication, is this just an adjunct along the way to help it? […] So, yes. That's what I'm just thinking, really. How extraordinary does this need to be seen as, almost?"	FG2, Consultant Psychiatrist
"We're not looking at what genes you have for a particular illness, this kind of genetic testing that needs specific consent and counselling, and sometimes to test for disorders, but yes, I mean it has to be clear where it's recorded, who sees and so on"	FG1, Consultant Psychiatrist
"I think it'll be worthwhile to let the patient know that it doesn't test their whole genetic profile. So that, that doesn't feel quite as potentially invasive […] I think making sure that you have the leaflets to describe how it works and what it works on. For instance, that little bit on the FAQ about answering the question about testing the whole genetic profile. That was reassuring."	ID2, White British Male
"What is the difference really between that [PGx] and seeing the information from the blood tests that we do now? I know it's genetics, but what really is the difference ethically?"	FG3, Mental Health Nurse
"It [PGx] is not that different to what our current processes are because we're asking for blood tests, we're asking for ECGs, we're doing physical health checks and it's also part of the conversation that we have every time around choice of antipsychotics so, I think it [PGx] fits well with our current routine practice."	FG1, Advanced Nurse Practitioner
**Theme 3: Timing PGx Right (it just depends)**	
"P: Maybe just consider where you introduce the idea of pharmacogenetic testing on the timeline of recovery of somebody experiencing psychosis. I think coming to them with this too soon, it could have negative effects. […] I: What do you think that those negative effects could be? P: It's basically the psychotic mind being quite active and being quite paranoid or suspicious or afraid and anxious. I think that getting worse, it could be considered a negative impact of introducing it too soon to a patient […] I might feel a little bit alarmed about anything to do with some genetic test. Because as you might know, a popular delusion that I think psychotic people experience is that their lives are being monitored."	ID2, White British Male
"Obviously you might say, "Oh [participant name], I'm taking your blood. Oh, [participant name] I'm taking a swab." But that could make someone's psychosis worse knowing know info like, "Oh, genetic testing." Because people have crazy ideas when they're ill […]. And hearing about genetic testing probably would've set me off worse."	ID12, White British Female

"For people who are already in the service you could implement it into the physical health clinic or something like that as well. Because that's people's routine [physical] checks."	FG3, Mental Health Nurse
"I probably feel like it wouldn't be suitable for someone that's in the middle of a psychotic episode because they wouldn't be able to process what's happening or what they're dealing with so […] it'd be a better either before, just on the onset of a psychotic episode or just after because the people around them would be able to explain to that person what had happened and then according to their understanding of what happened, they'd be able to accept that they've been put on a medication that's suited to their DNA. They'd be able to process that information better, […] it would be more like suitable for their needs at that time than in the middle of an episode."	ID6, British Asian Female
"I wouldn't want it to be implemented as a standard like everyone in EI has to sort of have this, you know, if they're going to have medication. I think there are those people who don't want to know certain things or, you know, don't ever want to try medication"	FG4, Mental Health Nurse
"I think not everybody might necessarily be fully vocal about the difficulties that they are having with their medication. But they might just rumble along with it, whatever, even though we do ask. It might be advocating more for those people, who might not necessarily speak up as much about some of the problems. It would be sad if it was available but not made available to everyone"	FG3, Consultant Psychiatrist
**Theme 4: PGx Concerns Me Because…**	
"They might be lacking capacity and obviously for them to consent for a test, they're going to have to have enough capacity to be able to do that and understand, really fully understand what it is about, what the implications are if they do the test against not doing the test so, there's the capacity issue."	FG1, Consultant Psychiatrist
"Some groups, like lower majority or racial minority groups may worry about, like, what is done with this information. They're worried about discrimination involved in the testing and keeping of data."	FG2, Psychotherapist / Family Therapist
"You create this system where it's so reliant on what this [PGx] report might say, that almost you're bypassed. You just become the prescriber of what that report says, you still have your own brain and thinking, and judgement, and you might disagree. […] It might point towards one drug, but you might have other information, or a nuanced conversation with that patient that said, 'no, actually, we're not going to go with that…'"	FG2, Consultant Psychiatrist
"I wonder if. Yeah, if it does take weeks, it's sort of delaying what we could sort of be doing early on. And I guess in the spirit of early intervention, we would want to get in there and sort of support people as quick as possible."	FG4, Clinical Psychologist
"I think there's a lot more complexity […] we just have to be careful that it doesn't lead to more of the biologicalisation of humans and stress […] John Read talks about how the biopsychosocial model has ended up the 'bio-bio-bio' model, and we just need to make sure that we've still got that broad view in early intervention, holistic care, of different things, so that it doesn't over-emphasise biology […]. I think there could be some real value in this [PGx], but I think there's some of my current concerns."	FG2, Consultant Psychologist
"I think that once it goes to an outside source, something like this, I think that ethically, I don't know. I just think that it's a little less ethical. You're treading on thin ice really as the potential for things to be misused, mis-stored, for accidents to happen, and I just think it would feel more comfortable for service users if	FG1, Support Worker

things happened inhouse and if we had our own lab set up, NHS labs that dealt with this."	
"I'm just wondering. Well, I don't know whether this would take up too much time. Because obviously a resource that we never have enough of is time"	FG3, Mental Health Nurse
"I would hold that concern, but it's pushed forward too quickly and that yeah, obviously, we don't get the resources that we need to be able to use it effectively."	FG4, Clinical Psychologist
"I don't have any concerns to be honest because after going through what it's meant to be for, I think it's just a much better and safer way to sort of find the right type of meds for patients to be on."	ID4, British Asian Female
**Theme 5: PGx in Practice**	
Sub-theme: PGx Education and Training	
"The devils in the detail, isn't it? As to the idea, I like, and I have no idea, [researcher name] - because I've not really read much about it, but I guess I'm just still would want far more information about what exactly we are looking at, and how accurate can it be, and what benefits have we seen before rolling it out."	FG4, Consultant Psychiatrist
"I just think somebody that's at least got a title or represent, or you know, the relevant education to give me that feedback rather than just somebody they've hired to keep costs low."	ID8, Mixed Black and White British Female
"I'm sure we'd need like someone training on the interpretation of the results, depending on what format they're given to us. That'll be useful to have someone talk through it and what it means and how accurate things are, so we can counsel patients correctly."	FG3, Consultant Psychiatrist
"I think with that, so some sort of, I know everyone loves it, a bit of a role play in terms of like how to have that conversation because there's something we've touched on quite a lot today about like you know, the informed consent and how to have that discussion"	FG4, Consultant Psychiatrist
"I think like face to face or video for prescribers really. Because you need to be able to ask questions on the day."	FG3, Consultant Psychiatrist
Sub-theme: Who is Involved in PGx?	
"I: And who would you want to communicate these [PGx] results to you? P: Probably somebody that I've built a rapport with, like as the care coordinator. Because that's somebody who I see a lot of. And I think when you're talking to somebody who's familiar to you, that tends to be more helpful."	ID2, White British Male
"I'd probably want to speak to my doctor in person to discuss the results with them in case I had any questions or like, I feel like it'd be best better to face that information in the presence of the doctor then If something worried me or like, made me anxious, then I'd be able to discuss that with somebody else present."	ID6, British Asian Female
"I need to know how it works, because […] someone's going to say, 'tell me everything about it' […] there's a professional responsibility to know what you're talking about. […] The more we're established on that [PGx] pathway, the more we'll need to know the technical answers […]. Or at least have access to that knowledge. […] I don't want my head to be scrambled by loads of science and data, but equally, you've got to be prepared for the fact that somebody might come and realistically say, 'I want you to tell me about this [PGx test]'"	FG2, Consultant Psychiatrist

"Care coordinators could have it as part of their requirements that they have to meet, to having that discussion with their patients when they see them"	FG3, Mental Health Nurse
"A good conversation about all the ins and outs with the carers because they're the people that are best placed as well to also be involved in decision making. Even though our young people are adults, they're not really in the best place at that time."	FG5, Carer, British Asian Female
"When I think about pharmacy, I think about the sort of… One of the words that comes to mind is safety, and consultation, and patient choice and all of those sorts of things. So, I wonder if it fits within the realm of pharmacy, or whether or not it's that kind of combination between pharmacy and medicine, really."	FG2, Mental Health Nurse
"In terms of the overview, of course we should be overviewing what's happening in that domain and also pharmacy, I'd say. Pharmacy would be good, yeah, so prescribers and pharmacy basically, I would say in terms of their overall responsibility and overviewing the process."	FG1, Consultant Psychiatrist
Sub-theme: Delivering and Reviewing PGx	
"Knowing the basics about what pharmacogenetic testing is and how it might be able to help improve the quality of care when you're on medication and […] knowing what it is to be able to find the right type of medication for you."	ID4, British Asian Female
"Partly comes down to the language and how these [PGx] things are sold, because I think a lot of the language that's being used today already, has the real potential of just rendering people numb. Then they're just going to go along with what the person in front of them tells them."	FG2, Consultant Psychiatrist
"If someone is worried or, you know, paranoid about like the government or different things and then we're trying to make a decision based on this genetic testing, I worry that we'd maybe say, if it was in guidance, we'd say well, it's the best interest decision."	FG4, Clinical Psychologist
"I believe that they all should be given choice whether they want to participate. I don't believe it should be something one gets enforced on."	ID10, British Asian Male
"The swab from the inside of the cheek. It's less awkward and it's less painful. I would just go for the path with least resistance"	ID2, White British Male
"I guess it depends how acute the presentation is and how quickly the person's needing some treatment. It might be two weeks before they'd meet [the psychiatrist or prescriber] then there's time to do tests and that would be great. A two-week window, we could manage that but sometimes if within the next 24 hours this person needs to be seen, we need to be thinking about treatment, what are we going to do, then it might be really difficult to be doing this [PGx]."	FG1, Mental Health Nurse
"Similar to how we already receive that sort of data, really, because for me, whether or not this is the result of someone's cholesterol profile […] or whatever it might be, genetic letters exist already on […] patient record systems. So, as long as there's the GDPR requirements that meet them, […] for me, it's making things as similar as they already are, so not to overwhelm practitioners to think."	FG2, Mental Health Nurse
"I'd feel fine with that if they modified it. But obviously they're going to let you know when stuff like that, the change in the medicine, the reason why it's changed due tests that you've done. The pharmacogenetic test, is it?"	ID5, Mixed Black and White British Female

"I'd probably say in the medical records because and then if you go to a different doctors or pharmacy, whatever, then they know that that you need this type of medication, or you've gone through this […] I think all the health professionals should know really."	ID5, Mixed Black and White British Female
"If we were to do pharmacogenetic testing, and we'd say, this information will go to your GP as most medical information sits at a central place, […] it goes to your GP to help us understand, your GP understand your overall health, have an overview of it all"	FG2, Mental Health Nurse
"I think I would feel comfortable if they were stored here and there. Stored with the GP and stored with early intervention psychosis too. Because the whole point of the testing is to get more accurate… a more effective treatment."	ID2, White British Male
"I think just touching base. So doing the [PGx] test, having the results, getting your prescription, and then having a contact point, in case you're having any adverse effects rather than, "Right, we'll ring you in a month." […] sometimes it might just be general information you need. […], so I just think maybe having like the care coordinator role could have been a little bit more accessible, I think. But I just think check ins just to make sure that the medication is going fine, and you know, you've not got any concerns because it's a reassuring thing as well."	ID8, Mixed Black and White British Female
